# Abdominal Fat and Sarcopenia in Women Significantly Alter Osteoblasts Homeostasis *In Vitro* by a WNT/****β****-Catenin Dependent Mechanism

**DOI:** 10.1155/2014/278316

**Published:** 2014-05-20

**Authors:** Francesca Wannenes, Vincenza Papa, Emanuela A. Greco, Rachele Fornari, Chiara Marocco, Carlo Baldari, Luigi Di Luigi, Gian Pietro Emerenziani, Eleonora Poggiogalle, Laura Guidetti, Lorenzo M. Donini, Andrea Lenzi, Silvia Migliaccio

**Affiliations:** ^1^Section of Health Sciences, Department of Movement, Human and Health Sciences, “Foro Italico” University of Rome, Largo Lauro De Bosis 15, 00195 Rome, Italy; ^2^Section of Medical Pathophysiology, Department of Experimental Medicine, Endocrinology and Nutrition, “Sapienza” University of Rome, Italy

## Abstract

Obesity and sarcopenia have been associated with mineral metabolism derangement and low bone mineral density (BMD). We investigated whether imbalance of serum factors in obese or obese sarcopenic patients could affect bone cell activity *in vitro*. To evaluate and characterize potential cellular and molecular changes of human osteoblasts, cells were exposed to sera of four groups of patients: (1) affected by obesity with normal BMD (O), (2) affected by obesity with low BMD (OO), (3) affected by obesity and sarcopenia (OS), and (4) affected by obesity, sarcopenia, and low BMD (OOS) as compared to subjects with normal body weight and normal BMD (CTL). Patients were previously investigated and characterized for body composition, biochemical and bone turnover markers. Then, sera of different groups of patients were used to incubate human osteoblasts and evaluate potential alterations in cell homeostasis. Exposure to OO, OS, and OOS sera significantly reduced alkaline phosphatase, osteopontin, and BMP4 expression compared to cells exposed to O and CTL, indicating a detrimental effect on osteoblast differentiation. Interestingly, sera of all groups of patients induced intracellular alteration in Wnt/**β**-catenin molecular pathway, as demonstrated by the significant alteration of specific target genes expression and by altered **β**-catenin cellular compartmentalization and GSK3**β** phosphorylation. In conclusion our results show for the first time that sera of obese subjects with low bone mineral density and sarcopenia significantly alter osteoblasts homeostasis *in vitro*, indicating potential detrimental effects of trunk fat on bone formation and skeletal homeostasis.

## 1. Introduction

Obesity and osteoporosis are important global health problems with high prevalence and impact on both mortality and morbidity [1, 2]. During the last decades both diseases have become a major health threat around the world, with age and female status increasing the risk of developing both obesity and osteoporosis [1, 2].

Interestingly obesity has been considered a protection factor against the development of bone loss and osteoporosis, likely for increased androgen aromatization to estrogens in postmenopausal obese women [3, 4] and, also, for a potential role of mechanical loading in regulating bone remodelling [5]. Recently, however, the belief that obesity is protective against osteoporosis has been questioned. Epidemiologic and clinical studies suggest that high level of fat mass might be a risk factor for osteoporosis and fragility fractures [6–8]. Moreover, adipose tissue functions as an endocrine organ by releasing several adipokines, which appear to modulate glucose and lipid metabolism, inflammation, appetite, and insulin resistance [9–11]. A physiological relevance of adipose tissue for skeletal health likely could reside in the role that an excess of proinflammatory cytokines, such as IL-6 and TNF-*α*, might play by interfering with bone cells homeostasis [12–15]. More recently, obesity has also been associated to sarcopenia, a condition characterized by progressive decline of muscle mass, quality, and strength [16, 17]. Thus, the crosstalk between fat and bone might play an important role as homeostatic feedback system in which adipokines and molecules secreted by bone cells represent the link of an active and functional bone-adipose-muscle axis [18–20], by mechanism(s) not fully clarified yet.

In addition, obesity is associated with a chronic low-grade inflammation as depicted by increased plasma levels of C-reactive protein (CRP), proinflammatory cytokines, and osteopontin [21, 22] as well as lower circulating levels of vitamin D (Vit D) [23]. Nevertheless, to date, few and conflicting data show a correlation among abdominal fat, Vit D, osteocalcin, inflammatory markers [24], and bone mineral density alterations in morbid obese women.

Since our group has recently demonstrated skeletal alterations in adult obese subjects and also an association of obesity with sarcopenia [8], aim of the present study was to investigate whether serum factors in obese and obese osteoporotic patients and/or sarcopenic patients could differently affect osteoblastic cells homeostasis* in vitro *and, likely altering skeletal health.

## 2. Materials and Methods

### 2.1. Subjects

Female subjects were selected in the Department of Experimental Medicine, Section of Medical Pathophysiology, Endocrinology, and Nutrition, of Policlinico Umberto I, Sapienza University of Rome. Chronic medical conditions, use of medications affecting bone metabolism, hormonal and nutritional status, vitamin D supplementation, recent weight loss, and prior bariatric surgery interventions were assessed as exclusion criteria. Patients were investigated for metabolic, inflammatory, and bone turnover markers and their sera used for culturing osteoblasts. Subjects were characterized by Dual Energy X-ray Absorptiometry (DEXA) and divided into different groups upon bone mineral density (BMD) and muscle mass. They were divided into different groups: the first group included obese women (body mass index, BMI >30) with normal bone mineral density and normal muscle mass (O); second group was formed by obese women with low bone mineral density for age (OO); another group included obese women affected by sarcopenia (OS); the last group included women with both low BMD and sarcopenia (OOS). A control group was also evaluated, which included healthy women with normal BMI and normal *T* score (CTL). Subjects' characteristics are reported in Table 1.

The research protocol was in accordance with the ethical requirements of the Helsinki Declaration and was approved by Ethical Committee of Policlinico Umberto I, Sapienza University of Rome. All subjects enrolled gave written informed consent.

### 2.2. Body Composition

The anthropometric measurements were taken following the procedures described in the anthropometric standardization reference manual [25]. Body weight was measured to an accuracy of 0.1 kg through a standard column body scale (SECA, Hamburg, Germany). Body height was determined using a rigid stadiometer (SECA, Hamburg, Germany) to an accuracy of 0.1 cm. BMI was calculated as body weight in kg/body height in m^2^.

Body composition (fat mass (FM) and fat-free mass (FFM)) was estimated by bioelectrical impedance analysis (BIA). The BIA measurement was performed on the right side of the body using 800-A and 50 kHz alternating sinusoidal current and a standard tetrapolar technique (Body Impedance Assessment, BIA 101 Impedance Analyzer, AKERN, Florence, Italy). Fat-free mass index (FFMI) was calculated as FFM in kg/(body height in m^2^).

### 2.3. Diagnosis of Obesity and Sarcopenia

Obesity in this female population was diagnosed as increased fat mass by 35%. Sarcopenia was diagnosed according to the definition by the European Working Group on Sarcopenia in Older People (EWGSOP), considering the reduction of muscle mass, the impairment of muscle strength, and physical performance [17]. FFM was considered depleted if a patient's fat-free mass (FFM) was <90% of their ideal FFM (iFFM). Ideal FFM was calculated in kg as the sum of 75% of ideal body weight 25% of overweight, as expressed by a BMI >25 kg/m^2^, considering that excess body weight includes not only body fat but also a certain amount of muscle mass [26]. Abdominal fat was evaluated and characterized by DEXA as previously described elsewhere [27, 28].

### 2.4. Cell Culture

Human SAOS-2 osteoblastic cells were cultured in Dulbecco's modified Eagle's medium (DMEM) supplemented with 2 mM L-glutamine, 100 U/mL penicillin/streptomycin, 10% fetal bovine serum (FBS), at 37°C and 5% CO_2_ in a humidified incubator. All buffers, media, and reagents were purchased from Euroclone. For patients sera treatments, cells were grown up to 70% confluence, starved overnight in a medium serum-free, and then incubated 24 hrs with medium supplemented with 20% patient serum. For RNA extraction and quantitative real-time evaluation, cells were treated with serum of single patients whereas for protein extraction and Western blot analysis and for immunofluorescence experiments, growing media were supplemented with a pull of sera of the same group.

### 2.5. RNA Isolation and Quantitative Real-Time PCR

For RNA extraction, cells were washed twice with 1X phosphate buffer saline (PBS, Euroclone) and total RNA was immediately isolated with Ambion RNA mini KIT (Ambion, Life Technologies) according the manufacturer's instructions. The purity and integrity of total RNA were monitored by electrophoretic analysis on denaturating agarose gel; ultraviolet spectrophotometry (Biorad) was used for RNA yield evaluation. Total RNA was treated as previously described [29]. Quantitative real-time PCR was performed in Abi Prism 7500 light cycler (Applied Biosystem) using Power SYBR Green PCR Master Mix (Applied Biosystem) as indicated by manufacturer. All primers were optimized as previously described [29]. Quantitative RT-PCR sample value was normalized for the expression of cyclophilin mRNA. The relative level for each gene was calculated using the 2^−ΔΔCt^ method [30] and reported as arbitrary units. In all experiments, each sample was analyzed in duplicate. Sequences of primers are given in Table 2.

### 2.6. Protein Extraction and Western Blot Analysis

Total proteins were extracted as previously described [29]. Twenty micrograms protein samples were separated in an SDS-polyacrylamide gel and transferred to a nitrocellulose membrane. Transfer was verified by Ponceau S staining. The membrane was blocked 60 min at room temperature with 5% nonfat dry milk (Cell Signaling) in T-TBS (tris-buffered saline plus Tween 20, 0.01%). Primary anti-LEF-1, anti-TCF-1 anti-c-myc, anti *β*-catenin, anti-GSK3*β* Ser9-phosphorilated, anti-totalGSK3*β*, and *β*-actin antibodies, diluted as indicated by manufacturers, were added overnight at 4°C. The membrane was washed three times with T-TBS and incubated for 60′ at room temperature with HRP-labeled anti-rabbit or anti-mouse in 5% nonfat dry milk. The membrane was then washed three times and antibodies were visualized using ECL Prime Western Blotting Detection Reagent (GE Healthcare). Quantitative analysis was performed using Imagequant TL Image analysis software (GE Healthcare); sample value was normalized for housekeeping gene *β*-actin and was reported as arbitrary units. All antibodies were purchased by Cell Signaling.

### 2.7. Immunofluorescence

For immunofluorescence (IF) analysis, cells were rinsed twice with cold PBS, fixed with 4% paraformaldehyde 15′ at room temperature, rinsed three times with PBS, permeabilized by incubation in 0.2% Triton in 3% bovine serum albumin (BSA), and rinsed accurately in PBS. Cells were incubated in blocking solution (6 ng/mL IgG from goat serum in PBS) for 30′ at room temperature and further incubated with anti-*β*-catenin (Cell Signaling) 1 : 200 in PBS overnight at 4°C. After three washes, cells were incubated with 1 : 500 anti-mouse Alexa Fluor (Molecular Probes) in the dark 45′ at room temperature. Cells were rinsed three times with PBS and slides were mounted with 70% glycerol in PBS. Images were acquired by Nikon Eclipse 600 microscope and Nikon CCD camera at 20x magnification.

### 2.8. Statistical Analysis

Data are expressed as means ± SE. Statistical significance between data points was determined using the two-tailed Student's *t*-test. A *P* < 0.05 was conventionally considered statistically significant.

## 3. Results

### 3.1. Patients Characteristics

Patients were characterized for anthropometric measurements, BMD, muscle mass as described in Section 2. Characteristics of different groups of subjects are depicted in Table 1 and described elsewhere [27]. Patients were similar for both age and body weight, as also described elsewhere [27].

### 3.2. Bone Cell Regulatory Factors in the Sera

No differences were found in hormones levels [27], beside a significant decrease of Vit D levels as previously published [23, 27]. To assess whether the sera of obese patients showed any altered balance of factors potentially affecting bone turnover, we collected sera of all subjects to culture cells and characterize modulation induced in osteoblasts activity* in vitro*.

### 3.3. Osteoblast Activity and Differentiation

To determine potential effects of the sera from obese patients on osteoblast homeostasis, cells were incubated with the sera of obese subjects to assess effect on cell differentiation and activity.

**Figure 1:**
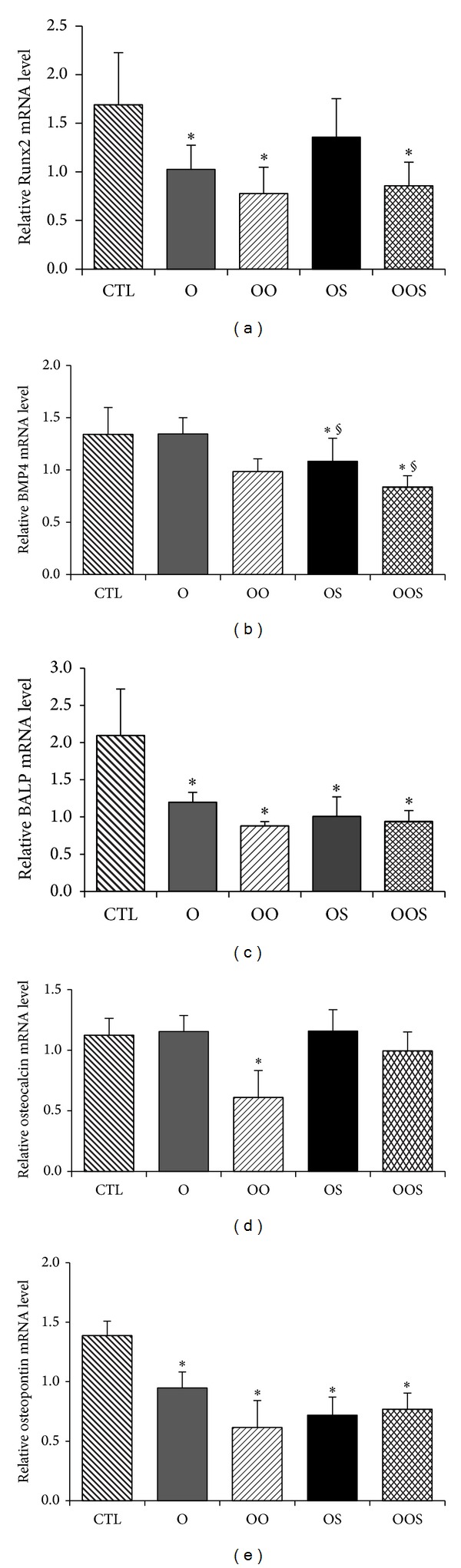
Expression of (a) Runx2, (b) BMP4, (c) ALP bone isoform d, (d) osteocalcin (OCN), and (e) osteopontin (OPN) in osteoblasts as measured by quantitative real-time PCR. Cells were grown in presence of sera from healthy normal body weight control individuals (CTL); obese patients (O); obese osteopenic patients (OO); obese sarcopenic patients (OS); obese sarcopenic osteopenic patients (OSO) as described in Section 2. Differences were considered significantly different when a *P* < 0.05 was obtained *P < 0.05 versus CTL; ^§^ *P* < 0.05 versus O.

Osteoblasts were cultured 24 hrs in presence of the sera obtained from individuals of the different experimental groups after which total RNA was extracted and quantitative RT-PCR performed to evaluate specific genes. Expression of Runx2, an essential transcription factor expressed during bone formation and osteoblast differentiation, was decreased in all experimental group, except that in the OS (*P* < 0.05, Figure 1(a)) while BMP4 mRNA expression was significantly decreased in cells grown in the sera of osteopenic patients (both OO and OOS) (*P* < 0.05, Figure 1(b)). The expression of BALP mRNA was significantly decreased in cells grown with the sera of all groups of obese patients compared to CTL (*P* < 0.05, Figure 1(c)), demonstrating an impairment of osteoblast activity. Interestingly, the expression of osteocalcin, the most important noncollagenic bone matrix protein, was significantly decreased only in cells exposed to sera of OO patients (*P* < 0.05, Figure 1(d)), strongly suggesting that the presence of different amount of muscle and adipose tissues can differently interfere with osteoblast biology. Finally, the expression of osteopontin (OPN), a highly phosphorylated bone matrix sialoprotein [31–35], was also significantly inhibited in osteoblasts incubated with sera of all groups of obese patients as compared to CTL (Figure 1(e), *P* < 0.05).

### 3.4. Wnt Targets mRNA/Protein

The following series of experiments were focused to further characterize the molecular mechanism(s) of action of the observed alteration of osteoblast homeostasis. The canonical Wnt/*β*-catenin pathway is essential for proliferation and survival of osteoblasts [36]. When the canonical Wnt ligands (i.e., Wnt1, 3a, and 8) bind to the cell-surface receptors, the intracellular protein disheveled (Dvl) is phosphorylated activating a cascade of intracellular events linked to the modulation of specific target genes expression [37, 38].

**Figure 2:**
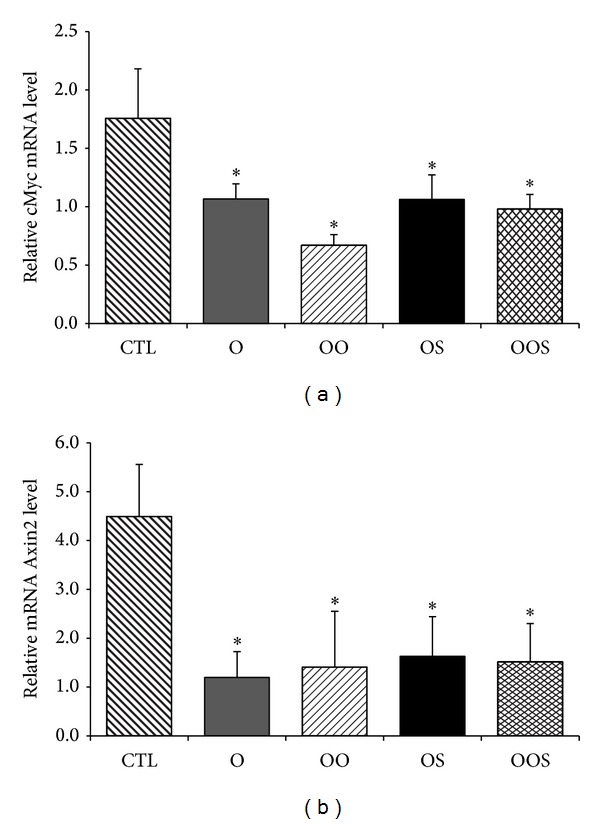
Expression of (a) cMyc and (b) Axin2 in osteoblasts grown as described in Figure 1. Differences were considered significantly different when a *P* < 0.05 was obtained *P < 0.05 versus CTL; ^§^ *P* < 0.05 versus O.

**Figure 3:**
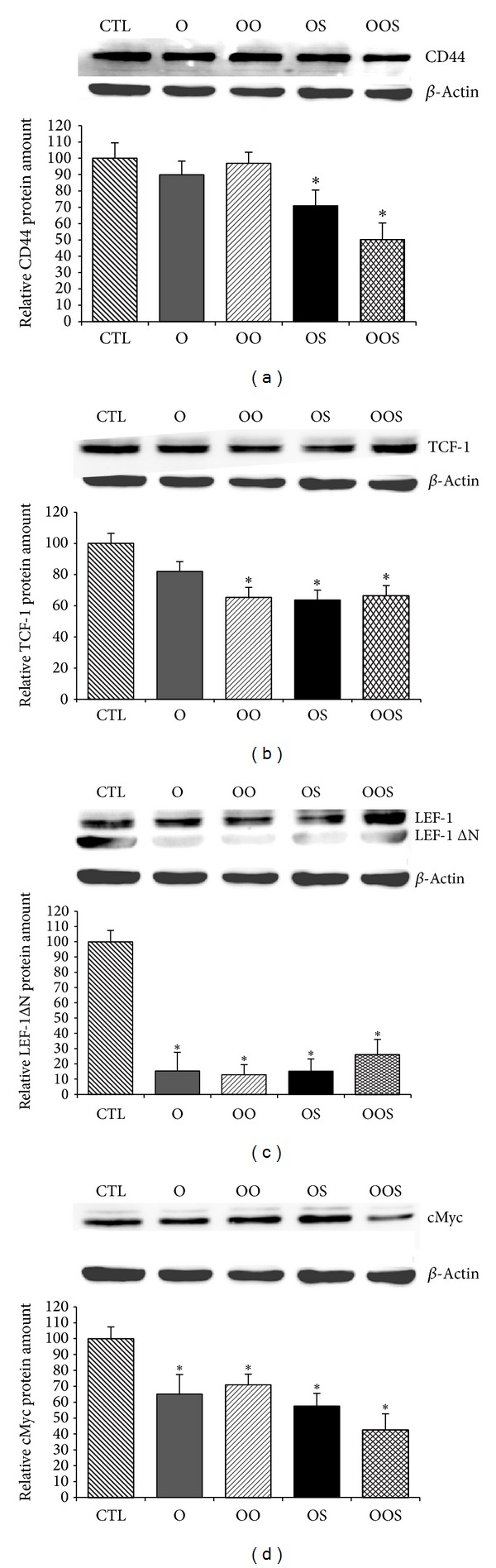
Western blot analysis of Wnt target genes protein expression (a) CD44, (b) TCF-1, (c) LEF-1ΔN, and (d) cMyc in Saos-2 osteoblastic cells grown as described in Figure 1. In each panel, upper row is the target gene and lower panel is loading control (*β*actin). Graph depicts protein quantification from Western blot evaluation. Bars represent mean ± SE of three different independent experiments performed in duplicate. *P < 0.05 versus CTL; ^§^ *P* < 0.05 versus O.

Thus, to evaluate whether canonical Wnt/*β*-catenin pathway was involved in the osteoblastic gene expression modulation exerted by obese sera, expressions of some specific target genes were evaluated by quantitative RT-PCR and Western blot analysis. Osteoblasts were grown and exposed to the human sera for 24 hrs as described above. Expression of cMyc and axin2 decreased in osteoblasts incubated in the presence of sera of O patients compared to CTL (*P* < 0.05) as shown in Figure 2. Likewise, the decrease of specific target gene expression was further confirmed by a reduction of protein expression of cMyc, LEF, CD44, and TCF1, as shown in Figures 3(a) and 3(b).

In particular, Wnt pathway inhibition was most significantly induced by exposure of cells to the sera of OS patients (CD44 inhibition almost 50%) as compared to the other groups, strongly indicating complex mechanism(s) underlying skeleton homeostasis in obese and sarcopenic subjects.

The expression of LEF1 protein was not significantly affected in cells exposed to the patients sera, whereas LEF1-ΔN was strongly inhibited in cells exposed to all obese groups (Figures 3(a) and 3(b)).

**Figure 4:**
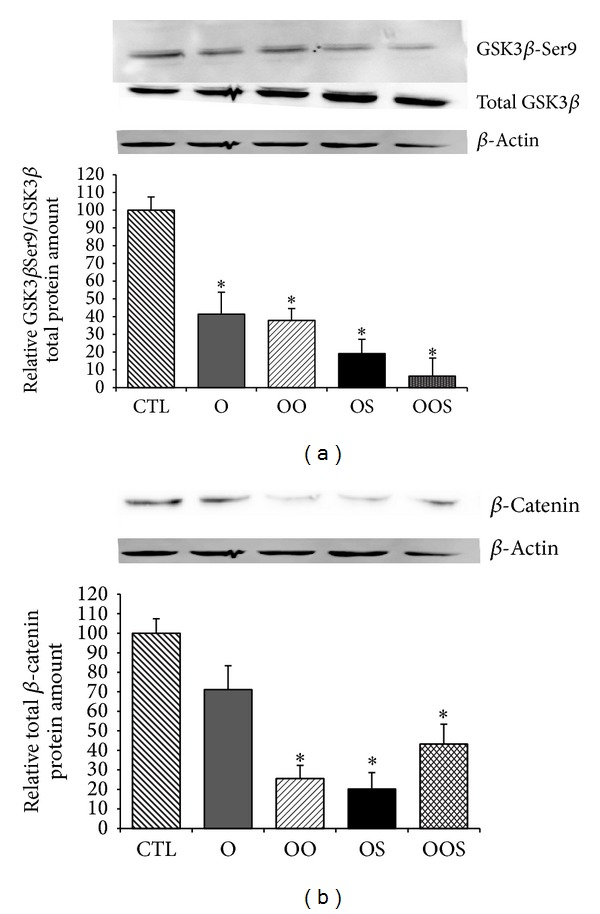
Western blot analysis of Wnt/*β*catenin pathway modulation. Expression of GSK3*β*Ser9 (upper panel, (a)) and total (middle panel, (a)) and total *β*catenin (upper panel, (b)) were evaluated in osteoblasts grown as described in Figure 1. Lower panel is the loading control (*β*actin). Quantifications of the data obtained from Western blot analysis are depicted in the graphs. Bars represent mean ± SE of three different independent experiments performed in duplicate. *P < 0.05 versus CTL; ^§^ *P* < 0.05 versus O.

**Figure 5:**
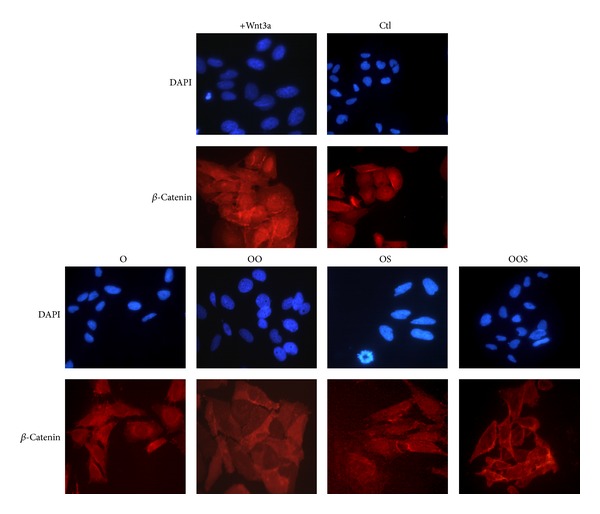
Characterization of *β*-catenin localization by immunofluorescence analysis in osteoblastic cells grown as described in Figure 1. A representative experiment of the three performed is shown in the figure. Magnification 40x.

To further investigate the potential mechanisms by which patient's sera modulate the Wnt/*β*-catenin pathway, GSK3*β*, a component of the *β*-catenin destruction complex and negative regulator of the Wnt pathway, was analyzed. In particular we checked both, by Western blotting analysis, phosphorylation at Ser-9 which inactivates GSK3*β* [39] and, by immunofluorescence, *β*-catenin nuclear translocation. A strong decrease of a ratio GSK3*β*-Ser9/total GSK3*β* was observed in osteoblasts upon exposure to the sera of all obese subjects (Figures 4(a) and 4(b)), demonstrating that the canonical *β*-catenin pathway is inactivated by the presence of nonphosphorylated (then active) GSK3*β*, and normal regulation within cells disrupted by obese subjects sera. Accordingly total *β*-catenin amount is decreased showing that protein is tagged for proteasomal degradation. Since nuclear localization of *β*-catenin is used as a marker of enhanced Wnt signaling, as expected, immunofluorescence analysis demonstrated a cytoplasmic localization of *β*-catenin in osteoblasts exposed to the sera of all obese subjects (Figure 5).

Taken together these* in vitro* data, showing the inhibition of canonical Wnt/*β*-catenin pathway, indicate that this signal transduction pathway is, likely, the intracellular signaling involved in the impairment of osteoblastic differentiation and activity exerted by obese sera.

## 4. Discussion

Our results demonstrate for the first time that the sera of obese osteoporotic and/or obese sarcopenic subjects can induce significant alterations in osteoblast homeostasis due to disruption in the Wnt/*β*-catenin differentiation-linked molecular pathway. In particular, sera of obese subjects blunt the canonical Wnt/*β*-catenin pathway inducing a downregulation of specific target genes with a consequent altered osteoblast differentiation and activity pattern.

Despite being a risk factor for cardiovascular and metabolic disease, for a long time obesity has been thought to protect against osteoporosis [3, 4], due to stresses from mechanical loading and from metabolic effects of estrogenic hormones secreted by adipocytes for testosterone aromatization [40]. However, recently, our group and others have demonstrated that increased visceral fat has negative effects on skeletal tissue health [7, 17, 27], indicating that further investigations were required to understand the complex interactions between bone and adipose tissue. Further recent evidences, in healthy premenopausal women, demonstrated that higher trunk fat mass was associated with inferior bone quality, as evidenced by markedly lower trabecular bone volume fraction, fewer and thinner trabeculae, lower trabecular stiffness, and higher cortical porosity [41]. Moreover the same study indicated that higher trunk fat mass is associated with higher bone marrow fat, lower trabecular bone volume fraction, and decreased bone formation* in vivo* [41]. Our results support the role of an altered bone formation in the skeletal alterations observed in obese subjects and further emphasize that the mechanism(s) of action underlying the observed alterations is likely due to a damaged pattern of osteoblastic differentiation which impairs cell activity. In addition, muscle mass appears to play a role in the interplay between adipose and bone tissue, likely by mechanical stimuli and also by myokines and other factors secretion [42]. Indeed, muscle is also source of IGF-1, known to be one of the factors which cooperate in the maintenance of skeletal health [43].In addition, lower levels of IGF-1 [27, 41] have been found in obese individuals (unpublished observation) and IGF-1 might play a pivotal role in the mechanism linking obesity to decreased bone density and bone quality [41], by mechanism due to altered osteoblast homeostasis. Indeed, the recent* in vivo* results, showing a weak increase in osteoclastogenic factors, go against the hypothesis that production of adipokines and inflammatory cytokines by adipose tissue lead to an increased bone resorption inducing a decrease in bone volume [28, 44].

More interestingly, we demonstrated that obesity induces detrimental effect on osteoblasts differentiation activity, suggesting that both bone resorption and bone formation are disrupted in obese patients. In particular, Runx2, which belongs to the RUNX family, is an essential transcription factor expressed during bone formation and osteoblast proliferation and differentiation and the blunted levels observed upon obese sera exposure further prove the osteoblast differentiation disruption [45]. Moreover, the decreased expression of all the osteoblastogenic differentiation and activity markers demonstrate the decreased osteoblast activity which further support the previously observed bone formation markers alteration in obese patients* in vivo* [41].

Wnt signaling pathway has emerged as a critical regulatory component of the control of bone formation and bone resorption [46]. When the canonical Wnt ligands (i.e., Wnt1, 3a, and 8) bind to the cell-surface receptors, the intracellular protein disheveled (Dvl) is phosphorylated inhibiting GSK3*β* from phosphorylating *β*-catenin in the cytoplasm. As result, *β*-catenin is stabilized and translocated into the nucleus where it establishes a transcriptional complex with T-cell factor (Tcf)/lymphoid enhancer-binding factor (Lef) to regulate expression of target genes [37, 38]. Our data demonstrate that canonical Wnt/*β*-catenin pathway is one of the molecular intracellular pathways involved in the gene expression modulation disrupted in the osteoblasts exposed to obese sera. Indeed, GSK3*β*, a key modulator of *β*-catenin activity, is repressed by phosphorylation at ser-9 and consequently *β*-catenin is slightly detectable and only in the cytoplasm. As a consequence, the *β*-catenin target genes are downmodulated and osteoblast differentiation and activity disrupted.

In regards of the Wnt molecular pathway, an interesting result is linked to the evaluation and characterization of Lef1 modulation. Lef1 contains two promoters that drive expression of a full-length protein (Lef1) and an N-terminally truncated isoform (Lef1-ΔN). Lef1 overexpression inhibits osteoblast maturation and Runx2 activity on the osteocalcin promoter* in vitro* [47, 48], but Lef1ΔN had the opposite effect [49]. Lef1-ΔN is present in mature osteoblasts and, despite the absence of the N-terminal high affinity *β*-catenin binding domain, retains the ability to weakly interact with *β*-catenin via second domain and to promote osteogenesis [50]. Interestingly, the sera of obese subjects can significantly blunt expression of Lef1-ΔN, strongly indicating an inhibition of osteoblast differentiation by an alteration of such molecular intracellular pathway.

Moreover, Wnt interacts with other signaling pathways in the bone cellular environment. One of these signals, IGF-1, enhances stabilization of *β*-catenin, triggering the molecular mechanism(s) leading to an increase of osteoblast differentiation and activity. Obesity is associated with decreased GH secretion and IGF-1 deficiency is associated with increased abdominal adipose tissue accumulation, decreased BMD [27, 42], and increased fracture risk as demonstrated by ours and other groups [41].

We understand that a limitation of our work is due to the fact that we have chosen a clonal osteoblastic cell line as our* in vitro* model system. However, it is well known that molecular or cellular mechanism(s) described in cellular model system is usually confirmed in primary cell lines, which we are evaluating as well.

Thus, our* in vitro* results further point out for a central role of IGF-1 and osteoblasts disruption in the mechanisms linking obesity to decreased bone quality.

Indeed, these results lead to the suggestive hypothesis that lower levels of IGF-1 in subjects affected by an increased abdominal adipose tissue accumulation could contribute to the blunted levels of *β*-catenin signaling in the osteoblasts leading to a disruption of normal differentiation pathway. Further investigations are needed to clarify and characterize the complex link between adipose-bone and muscle tissues and identify other potentially involved molecules, beside IGF-1, linked to the observed altered Wnt/*β*-catenin pathway.

## Figures and Tables

**Table 1:** Anthropometric data of the subjects involved in the study.

	CTL (10)	O (31)	OO (9)	OS (36)	OSO (15)
Mean age	43.4 ± 10.5	49.0 ± 10.0	62.0 ± 9.0	49.7 ± 13.2	62.0 ± 11.0
BMI	23.3 ± 1.67	37.9 ± 5.92	35.6 ± 8.0	38.5 ± 7.7	34.2 ± 4.9

CTL: control group, O: obese subjects, OO: osteoporotic obese subjects, OS: sarcopenic obese subjects, OSO: sarcopenic osteoporotic obese subjects. Data are expressed as mean ± standard deviation.

**Table 2:** Primers used in the experimental conditions.

Gene	Accession number	Forward primer	Reverse primer
BALP	NM000478	GGCTCCAGGGATAAAGCAGGT	AGTGTCTCTTGCGCTTGGTCT
Osteocalcin	NM199173	ATGAGAGCCCTCACACTCCTCG	GTCAGCCAACTCGTCACAGTCC
RUNX2	NM001024630	TTCTGCCTCTGGCCTTCCAC	TGGAGAAGCGGCTCTCAGTG
BMP4	NM001202	GCTTGTCTCCCCGATGGGATT	CTCGGGATGGCACTACGGAA
OPN	NM001251830.1	GCAGACCTGACATCCAGTACC	GATGGCCTTGTATGCACCATTC
cMyc	NM002467.4	TTCTCTCCGTCCTCGGATTCT	TTGTTCCTCCTCAGAGTCGCT
Axin2	NM004655.3	GACAGGTCGCAGGATGTCTG	TGTGCTTTGGGCACTATGGG
Cyclophilin	NM021130	GTCAACCCCACCGTGTTCTT	CTGCTGTCTTTGGGACCTTGT
